# *In situ* Pulmonary Artery Thrombosis: A Previously Overlooked Disease

**DOI:** 10.3389/fphar.2021.671589

**Published:** 2021-07-08

**Authors:** Yunshan Cao, Chao Geng, Yahong Li, Yan Zhang

**Affiliations:** ^1^ Department of Cardiology, Gansu Provincial Hospital, Lanzhou, China; ^2^ Tianjin Key Laboratory of Retinal Functions and Diseases, Tianjin Branch of National Clinical Research Center for Ocular Disease, Eye Institute and School of Optometry, Tianjin Medical University Eye Hospital, Tianjin, China

**Keywords:** pulmonary thromboembolism, in situ pulmonary thrombosis, deep venous thrombosis, chronic thromboembolic pulmonary hypertension, risk factors, pathogenic mechanism

## Abstract

Pulmonary thromboembolism (PTE) is the third leading cause of death in cardiovascular diseases. PTE is believed to be caused by thrombi detached from deep veins of lower extremities. The thrombi travel with systemic circulation to the lung and block pulmonary arteries, leading to sudden disruption of hemodynamics and blood gas exchange. However, this concept has recently been challenged by accumulating evidence demonstrating that de novo thrombosis may be formed in pulmonary arteries without deep venous thrombosis. On the other hand, chronic thromboembolic pulmonary hypertension (CTEPH), a subtype of pulmonary hypertension, could have different pathogenesis than traditional PTE. Therefore, this article summarized and compared the risk factors, the common and specific pathogenic mechanisms underlying PTE, in situ pulmonary artery thrombosis, and CTEPH at molecular and cellular levels, and suggested the therapeutic strategies to these diseases, aiming to facilitate understanding of pathogenesis, differential diagnosis, and precision therapeutics of the three pulmonary artery thrombotic diseases.

## Introduction

Pulmonary thromboembolism (PTE) is the third common cardiovascular disease following myocardial infarction and ischemic stroke (Goldhaber and Bounameaux, 2012). PTE is caused by the thrombi that disrupt pulmonary circulation, leading to pulmonary hypertension (PH), right heart failure (Moorjani and Price, 2013; Agnelli and Becattini, 2015), and even death (Prabhu and Soukas, 2017). The thrombi were initially thought to originate from deep veins in lower extremities, pelvis or right heart, travel with systemic circulation to pulmonary vasculature, and suddenly or recurrently block pulmonary arteries. However, this concept has recently been challenged by the evidence demonstrating that *de novo* thrombosis may be formed in pulmonary arteries without deep vein thrombosis (DVT) in lower extremities.

## The Origin of “Pulmonary Embolism”

More than a century ago, a German physician and scientist Rudolf Virchow proposed that blood clots in the leg could travel to the lung and cause pulmonary embolism (PE), because he discovered at autopsy that the emboli in the lung and the leg usually coexisted. He also performed an experiment showing that foreign bodies in deep veins can be found in pulmonary arteries (Virchow, 1856). Therefore, he coined the terms “PE” and “DVT” (Bagot and Arya, 2008). From then on, embolus from peripheral venous system, such as lower extremities and pelvis, has been deemed the predominant cause of pulmonary artery obstruction. However, from the contemporary perspective, Dr. Virchow’s proposal might be biased and excluded the possibility of *de novo* thrombosis in pulmonary vessels. Based on Virchow’s proposal, PE and DVT are disparate manifestations of the same disease, both belonging to venous thromboembolism (VTE) (Sakuma et al., 2009). In addition, chronic thromboembolic pulmonary hypertension (CTEPH), the group IV in the WHO classification of PH, has been deemed as a chronic stage of PTE, and thus designated as post-PTE syndrome (Huisman et al., 2018; Mullin and Klinger, 2018). However, the conclusive evidence proving the causal relationship between DVT and PTE as well as that between PTE and CTEPH is lacking, and the origin of thrombi in CTEPH is controversial (Kantake et al., 2013; Huisman et al., 2018).

## *In situ* Pulmonary Thrombosis: Pulmonary Thromboembolism Without Deep Vein Thrombosis

In recent years, multiple lines of evidence have indicated the possibility of generating *de novo* thrombus in pulmonary arteries without DVT in lower extremities. For example, Benns et al. (2014) conducted a retrospective study of incipient PTE (within 72 h post admission) in post-traumatic patients, and found that 84.2% of the PTE patients had no DVT. Similarly, Paffrath et al. (2010) investigated 7,937 post-traumatic hospitalized patients, with only 146 of them developing VTE; furthermore, 37% of these VTE patients were not accompanied with DVT. In addition, among the 11,330 patients that had received post-traumatic services, 2,881 were monitored by duplex sonography, and the results demonstrated the greater prevalence of PTE without DVT than that of PTE with DVT (Van Gent et al., 2014). Velmahos et al. (2009) performed computed tomographic venography of the pelvic and lower extremity proximal veins and computed tomographic pulmonary angiography in post-traumatic patients, and they found that few PTE cases were accompanied with DVT. One possible explanation of these observations is the complete dislodgment of the deep vein thrombus and the subsequent obstruction of the main branch of pulmonary artery. Nevertheless, Van Gent et al. (2014) performed duplex sonography in 12 patients with PE and DVT, and found that 83% of these patients had residual clot in lower extremities. In addition, the autopsies revealed that 59% the patients with PE had a DVT at the time of death (Lindblad et al., 1991). Therefore, although we cannot exclude the possibility that the entire deep vein thrombus falls off and causes PTE, in the majority of cases, only a portion of the clot is dislodged to form thrombus in pulmonary artery. More interestingly, the alternative explanation may be that PTE and DVT are distinctive pathologies or occur simultaneously rather than one leading to another. This explanation has been supported by a considerable amount of literature (Knudson et al., 2011; Van Gent et al., 2014; Cha et al., 2015; Brown et al., 2018). Therefore, to distinguish from the PTE associated with DVT, we propose the term “*in situ* pulmonary artery thrombosis (*in situ* PAT)” to describe the pathology of *de novo* thrombosis in proximal (main, lobar, and segmental arteries), distal (segmental, mid-segmental, and sub-segmental arteries, down to small pulmonary arteries of 2–5 mm in diameter), and micro (microvasculature of 0.1–0.5 mm in diameter) pulmonary arteries (Burman et al., 2016; Madani et al., 2017).

## The Risk Factors of Pulmonary Thromboembolism Associated With Deep Vein Thrombosis and of *in situ* Pulmonary Artery Thrombosis

**FIGURE 1:**
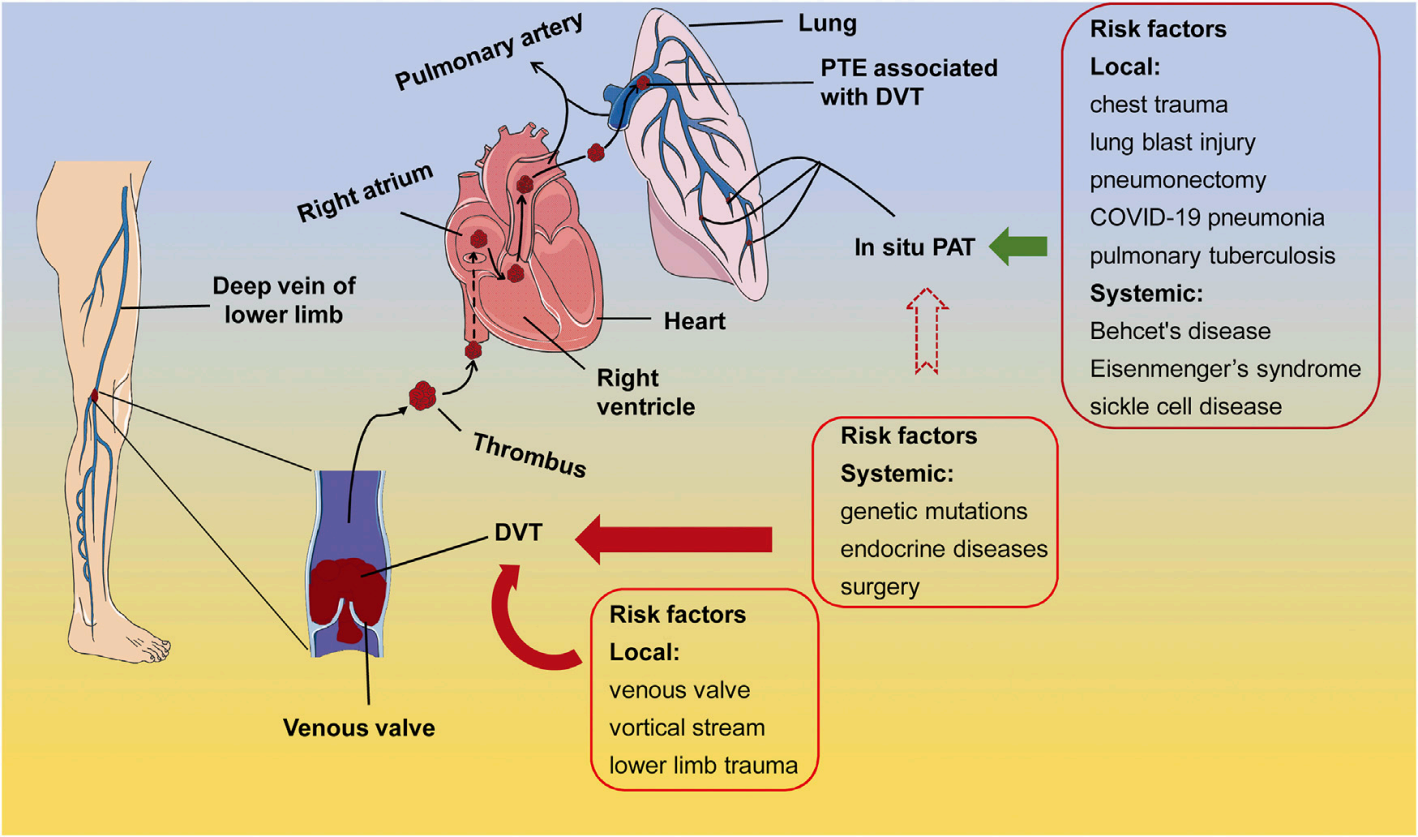
The risk factors of PTE associated with DVT and *in situ* PAT. In the majority of cases, the systemic susceptible conditions, such as genetic mutations, endocrine disorders, and surgery, as well as the local conditions, such as anatomical and hemodynamic characteristics and trauma, elicit thrombus formation at the venous valves in lower extremities. After shedding from the venous valves, the thrombus travels through circulation to block either the main body or branches of pulmonary artery, leading to the PTE associated with DVT (arrows in dark red). On the other hand, pulmonary diseases, lung damage, and immunological, congenital, and hematological systemic diseases may cause *in situ* PAT (arrow in dark green). It is also possible that *in situ* PAT is formed under the susceptible systemic conditions of DVT, however, direct evidence is lacking (arrow in dark red dotted lines). PTE, pulmonary thromboembolism; DVT, deep vein thrombosis; *in situ* PAT, *in situ* pulmonary artery thrombosis.

It used to believe that PTE developed from DVT, and they belonged to the consecutive processes of venous thrombosis. However, Van Gent et al. (2017) assessed the risk factors of PTE and DVT in adult patients with traumatic injuries and suggested that PTE and DVT are clinically distinct events with independent risk factors and occur at different time post traumatic injuries. The PTE derived from DVT is associated with circulatory and anatomical susceptible characteristics in lower extremities; whereas the PTE concurring with DVT may be related to vascular dysfunctions caused by systemic disorders and stress, including genetic factor-related factor V Leiden mutation, endocrine dysregulation, obesity, and surgery (Huisman et al., 2018) (Figure 1).

On the other hand, lung trauma and congenital or acquired abnormalities in lung structures are considered high risk factors of *in situ* PAT (Van Gent et al., 2014; Fletcher-Sanfeliu et al., 2020) (Figure 1). Experimental studies have shown that chest trauma may induce focal inflammation and dysfunction in pulmonary vascular endothelial cells, eliciting *in situ* PAT (Knudson et al., 2011; Brakenridge et al., 2013) (Figure 1). For instance, in a mouse model of unilateral thoracic contusion, eccentric fibrin aggregates on and platelets adhere to the endothelial cells aligning inner surface of pulmonary arteries after chest trauma (Brown et al., 2018), initiating *in situ* PAT (Schutzman et al., 2018). Additionally, *in situ* PAT is associated with the aberrant pulmonary structures, such as pulmonary artery stump following pneumonectomy (Kim et al., 2005; Kwek and Wittram, 2005), compensatory dilation and sheer stress at the proximal pulmonary artery caused by congenital cardiovascular defects and the resulting PH (Celestin et al., 2015), and pulmonary tuberculosis-destoryed lungs (Cha et al., 2015), as well as with the systemic predisposing conditions, including Behcet’s disease (Yilmaz and Cimen, 2010), Eisenmenger’s syndrome (Silversides et al., 2003; Broberg et al., 2007), sickle cell disease (Mekontso Dessap et al., 2011), and other systemic diseases (Porembskaya et al., 2020) (Figure 1). In general, local factors in the lung or lower limbs may contribute to thrombosis in the corresponding organ; whereas systemic factors such as autoimmune diseases and inflammation could lead to both DVT and PAT (Figure 1).

## The Risk Factors of Chronic Thromboembolic Pulmonary Hypertension

CTEPH is the mechanical obstruction of pulmonary vessels caused by thromboembolism, which leads to pulmonary vascular remodeling and progressive PH (Yandrapalli et al., 2018). The incidence of CTEPH after symptomatic PTE is reported between 0.1 and 9.1% (Lang and Madani, 2014). However, it is difficult to determine the actual CTEPH incidence, due to the absence or non-specificity of early symptoms and signs of this disease (Klok et al., 2018; Konstantinides et al., 2019). Moreover, a significant portion of CTEPH patients lack a history of acute PTE or DVT (Galie et al., 2015), thus *in situ* PAT has been considered as the cause of CTEPH in these cases (Egermayer and Peacock, 2000).

**FIGURE 2:**
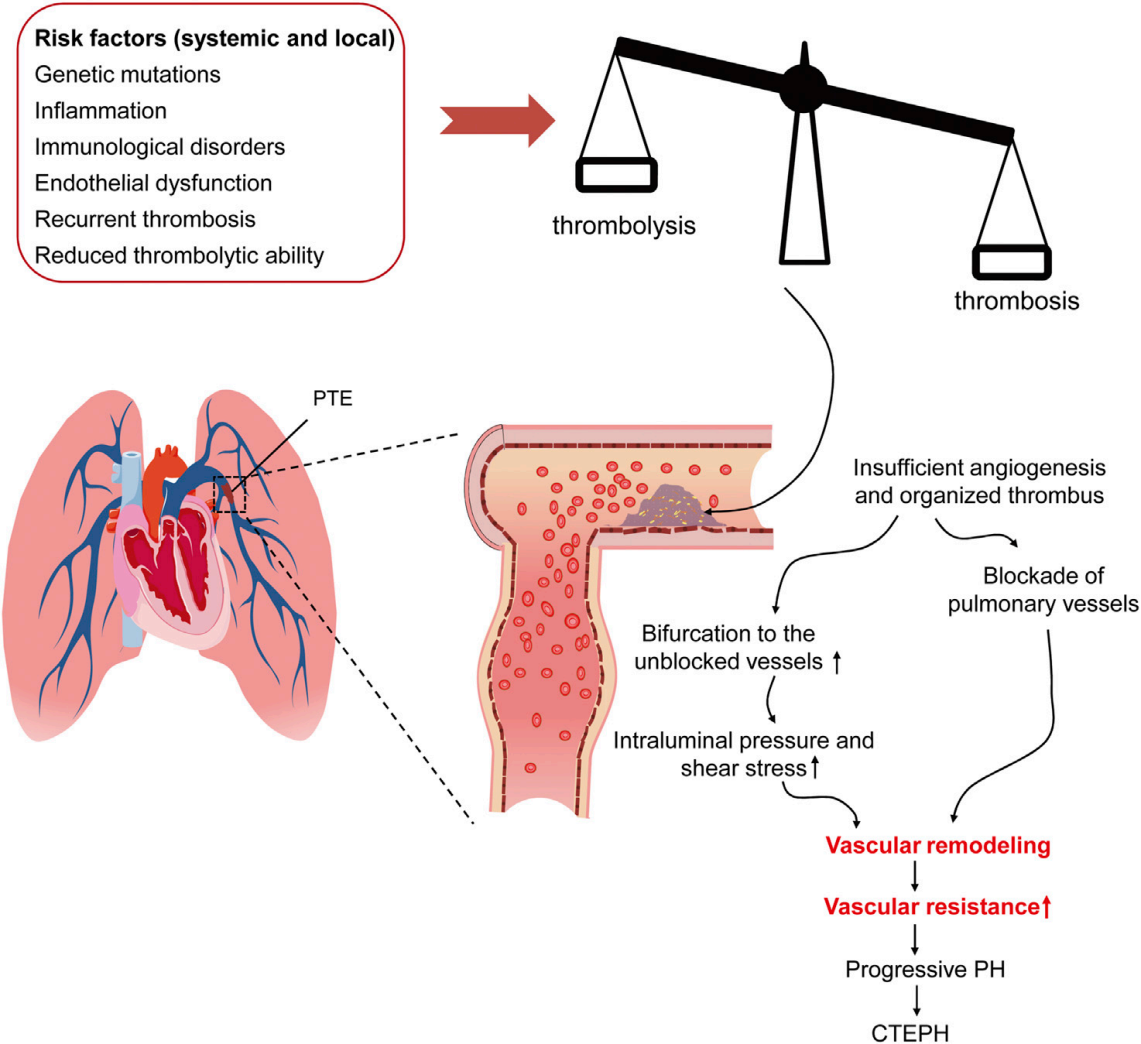
The risk factors of and pathogenic mechanism underlying CTEPH. The pathological conditions, including genetic mutation, inflammation-associating endothelial dysfunction, recurrent thrombosis, and reduced thrombolytic activity, result in the imbalance between thrombosis and thrombolysis, which causes insolubility and organization of the thrombi in pulmonary arteries. As the thrombi block the pulmonary arteries, the pulmonary circulation is diverted to the unblocked ones, where intravascular pressure and shear stress is consequently elevated. Both the thrombus-mediated blockade of pulmonary arteries and the diverted blood-mediated elevation in mechanical stress lead to vascular remodeling, increased pulmonary vascular resistance, progressive PH. CTEPH, chronic thromboembolic pulmonary hypertension; PTE, pulmonary thromboembolism; PH, pulmonary hypertension.

The initiating factors of CTEPH remain controversial, although several candidates have been proposed, including incomplete resolution and then organization of thrombi in pulmonary arteries following acute PTE, single and recurrent silent PTE derived from deep veins in lower extremities, and the thrombi formed at pulmonary arteries (Kantake et al., 2013; Huisman et al., 2018) (Figure 2). Therefore, the risk factors of CTEPH overlap with those of PTE, DVT, and *in situ* PAT. For the CTEPH patients with a history of acute or recurrent PTE, anatomical susceptible conditions in lower limbs, heamatological and endocrine disorders, and surgical interventions would be the risk factors of these patients; whereas tramatic injury and anatomical aberration in lung, and systemic diseases incurring inflammation, hypoxia, and abnormalities in pulmonary arteries subserve the risk factors for the CTEPH patients with no history of PTE and DVT. Moreover, ethnicity has been shown to play a role in determining CTEPH characteristics. Chausheva et al. (2019) compared the clinical parameters, hemodynamics, inflammatory factors, and thrombi of the CTEPH patients undergoing pulmonary endarterectomy in Austria and Japan, representing the people of European and Asian origins, respectively. The differences in the physiological parameters, including body size, lung vital capacity, cardiac output, and blood tests, are within expectation. Furthermore, the study revealed equipoise gender affliction, a prevalent history of PE, and a phenotype of metabolic syndrome in Austrian patients with CTEPH. Moreover, plasma levels of C-reactive protein and myeloperoxidase were significantly elevated in Austrian patients as compared to Japanese counterparts, implicating the proinflammatory pathogenesis of CTEPH in Australian patients. Consistent with these findings, the thrombi in Austrian patients occupied larger areas and exhibited a more inflammatory and fresh phenotype than Japanese patients (Chausheva et al., 2019). Additionally, abnormalities of fibrinogen (Morris et al., 2009), elevated factor VIII, antibodies to phospholipid, splenectomy, chronic inflammatory disease, ventriculoatrial shunt, hypothyroidism, and cancer (Bonderman et al., 2003; Jaïs et al., 2005; Kim and Lang, 2012) have been reported as risk factors of CTEPH (Figure 2).

Although CTEPH can present as acute attack, the majority of CTEPH patients manifest as chronic PH. For the CTEPH patients with a history of acute PE, a trigger, such as surgery, usually exist, and the clinical manefestations during the episode of acute PE include acute attack of shortness of breath and hypoxemia, sometimes the triad including chest pain, hemoptysis, and shortness of breath may be observed, and severe patients may have acute right heart failure (Klok et al., 2020). On the other hand, the CTEPH patients without PE present the similar clinical characterisctics as PH, such as exertional shortness of breath. It is of note, however, that when fresh thrombi in asymptomatic CTEPH generate salient symptoms and signs, CTEPH is often mistakenly diagnosed as acute PTE (Klok et al., 2018). Under such circumstance, the origin of thrombus, the presence of fresh intraluminal thrombus, the evidence of organic mural thrombus, the enlarged and hyperplastic right heart, and the formation of systemic collateral branches are of great significance for differential diagnosis between acute PTE and CTEPH.

## Common Pathogenic Mechanisms Underlying Thrombosis

### Vascular Endothelial Cell Injury

The vascular endothelial cells express anticoagulant and vasodilatory factors, thereby preventing blood coagulation and platelet adhesion as well as promoting vessel dilation and fibrinolysis (Gresele et al., 2010). Specifically, endothelial cells produce thrombomodulin and activate anti-coagulant protein C to maintain anti-coagulation. Moreover, endothelial cells express heparin sulfate and tissue factor pathway inhibitor (TFPI) to boost the activities of anti-thrombin III and fibrinolytic factors, respectively (Van Hinsbergh, 2012). Endothelial cells also generate nitric oxide, prostacyclin, and ectonucleotidase CD39, which can prevent platelet activation and inhibit coagulation (Furie and Furie, 2008). When the defense of vascular endothelial cells against thrombus is dismantled, thrombosis occurs.

**FIGURE 3:**
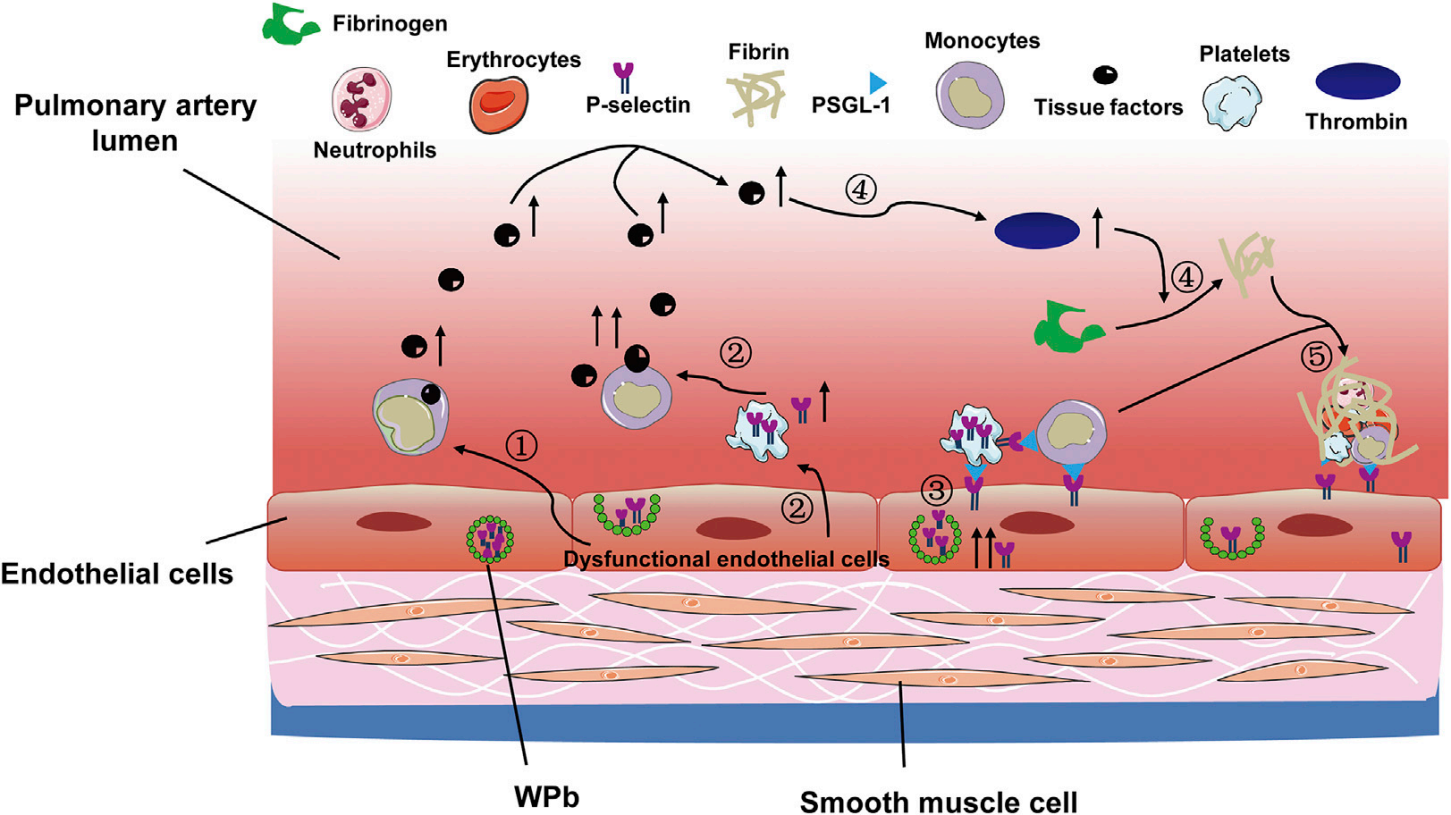
The coagulation cascade activated following pulmonary vascular endothelial cell injury. The pulmonary vascular hypoxic and inflammatory scenario leads to the following events: 1) endothelial cell injury and upregulates TF expression on monocytes; 2) the injuried endothelial cells promote platelets to release P-selectin, which further increases TF production; 3) the endothelial cells per se release P-selectin, which adheres to platelets and leukocytes, facilitating their deposition onto the endothelia; 4) the increased production of TF enhances thrombin production, which promotes conversion of fibrinogen to fibrin; 5) the deposited leukocytes and platelets are entangled with fibrin, leading to *in situ* pulmonary thrombosis. TF, tissue factor; WPb, Weibel-Palade bodies; PSGL-1, P-selectin glycoprotein ligand 1.

On the other hand, the vascular endothelial cells can be activated by injuries, and the activated endothelial cells stimulate leukocytes to express tissue factor (TF) and promote release of von Willebrand Factor and P-selectin in Weibel-Palade bodies (Figure 3). The released factors are rapidly transferred to the intraluminal surface and binds to P-selectin glycoprotein ligand 1 on platelets and leukocytes. Besides, the expression of P-selectin is also upregulated in platelet α granules (López and Chen, 2009; Schutzman et al., 2018; Schutzman et al., 2019). P-selectin could further upregulate TF expression in leukocytes, particularly in monocytes, thus forming a positive feedback regulatory loop (Figure 3). In addition, the endothelial injury renders the collagen and TF originally beneath endothelial cells exposed to blood circulation (Furie and Furie, 2008). Collagen triggers platelet accumulation and activation. The exposed subendothelial TF and the upregulated TF on leukocytes together mediate production of thrombin and catalyze conversion of fibrinogen to fibrin (Celi et al., 1994). The fibrin promotes deposition of monocytes and neutrophils, eventually leading to thrombosis (Yan et al., 2000) (Figure 3).

### Hypercoagulable State

The balance between anticoagulants and procoagulants maintains homeostasis of blood flow (Aird, 2007). When the balance is tilted by congenital and/or acquired disorders, the reduction of anticoagulants and/or the elevation of procoagulants generates the hypercoagulable state. The congenital disorders include congenital deficiency in thromboplastin inhibitor, such as antithrombin, protein C, and protein S, prothrombin-induced protein C resistance (Crous-Bou et al., 2016), and factor V Leiden gene mutation (Martinelli et al., 1998).

The acquired disorders are comprised of age, cancer, pregnancy, oral contraceptives, hormone replacement therapy, and obesity. In specifics, during the process of senescence, vascular elastic fiber degenerates (Chattopadhyay et al., 2011); levels of vascular wall-associated anticoagulants decrease, and those of procoagulants increase (Lowe et al., 1997; Esmon, 2009).

Cancer cells induce the hypercoagulable state through activating host coagulation system, such as upregulating expression of haemostatic factors, pro-inflammatory factors, and adhesion molecules, as well as directly adhering to host cells (Soo Hoo, 2013; Falanga et al., 2017). Indeed, studies have shown that the activity of circulating microparticle-associated TF, which can induce coagulation and thrombosis, is augmented in the blood of the patients with malignancies (Tesselaar et al., 2007). In addition, cancer treatments with either cytotoxic drugs such as cisplatin (Seng et al., 2012) or targeted chemotherapies including monoclonal antibodies to epidermal growth factor receptor (Petrelli et al., 2012) and tyrosine kinase inhibitors of vascular endothelial growth factor receptor (Qi et al., 2014) have been reported to incur higher incidence of venous and arterial thrombotic events as compared to the treatment modalities not involving these cytotoxic or targeted drugs. The underlying mechanism could be due to the off-target effects of these drugs, for instance, the non-specific cytotoxicity of cisplatin to and the impact of growth factor deprivation exerted by the tyrosine kinase inhibitors on vascular endothelial cells. Alternatively, these drugs may decrease anticoagulants and increase procoagulants, hence tipping the balance towards coagulation. Finally, the anticancer drugs may directly or indirectly activate platelets (Grover et al., 2021). All of these mechanisms may promote thrombosis under malignancies. There were also case reports showing that administration of immune checkpoint inhibitors, such as monoclonal antibodies to programmed cell death-1 or its ligand and to cytotoxic T lymphocyte-associated antigen 4, is associated with venous and arterial thrombosis (Boutros et al., 2018), however, the results of a systematic review demonstrated that the thrombotic events associated with the immune checkpoint inhibitors are relatively rare in the patients with advanced cancer (Solinas et al., 2020).

During pregnancy, the levels of coagulation factor V, VII, VIII, IX, X, XII, and von Willebrand Factor increase; while the level of anticoagulant protein S, plasma fibrinolytic activity, and acquired protein C resistance reduce (Brenner, 2004; Holmes and Wallace, 2005). What’s more, pregnancy, oral contraceptives, and hormone replacement therapy trigger venous thrombosis through elevating estrogen levels and enhancing thrombin production (Ohashi et al., 2003).

In obese patients, the proinflammatory cytokines secreted by hypertrophic and hyperplastic adipocytes result in chronic low-grade inflammation, which in turn activates prothrombotic signals and upregulates plasminogen activator inhibitor 1, contributing to the occurrence of VTE (Samad et al., 1996; Vandanmagsar et al., 2011; Blokhin and Lentz, 2013).

### Inflammation

At early stage of inflammation, the endothelial cells recruit inflammatory cells to the damaged or infected site for defense and repair (Gresele et al., 2010). The upregulated P-selectin on the endothelial cells binds to the P Selectin Glycoprotein Ligand 1 (PSGL-1) on the leukocytes, facilitating leukostasis and extravasation (Myers et al., 2003; Wakefield and Henke, 2005). Moreover, inflammation may also activate endothelial cells and promote their switch to the pre-thrombotic and anti-fibrinolytic phenotype. Then the activated endothelial cells upregulate the expression of adhesion molecules, facilitating their adhesion to monocytes and platelets (Van Hinsbergh, 2012). In addition, inflammation activates platelets to secrete modulators, including adenosine triphosphate, adenosine diphosphate (ADP), serotonin, cyclooxygenase, and thromboxane, thereby enhancing vasoconstriction and platelet aggregation (Shi and Morrell, 2011). What’s more, the activated platelets bind to PSGL-1 and CD40 on the endothelial cells and leukocytes through the upregulated expression of P-selectin and the ligand of CD40, respectively, promoting the formation and deposition of platelet-leukocyte complex (Wakefield et al., 2008; Nurden, 2011). Finally, proinflammatory cytokines can stimulate monocytes to produce TF, which, as mentioned above, activates coagulation cascade (Aksu et al., 2012).

In addition, microparticles are small membranous vesicles containing bioactive molecules and participating in intercellular communications. Microparticles are released by different types of activated or apoptotic cells, including endothelial cells, platelets, and leukocytes (Yan et al., 2017; Zarà et al., 2019) The production of microparticles is increased under the conditions of inflammation, infection, and malignancy (Ardoin et al., 2007). The microparticles promote coagulation through three mechanisms. One is to directly activate coagulation cascade via expressing TF and phosphatidylserine. Another mechanism is that the endothelial cell-deriving microparticles carry P-selectin that can bind to PSGL-1 on monocytes and platelets, thereby facilitating the deposition of the latter two types of cells onto the vessel walls. Thirdly, the microparticles also express the biomarkers of leukocytes and platelets, which promotes cell-cell interaction and augments coagulation (Wakefield et al., 2008).

### Hypoxia

Hypoxia is the stimulating factor of thrombosis. Under hypoxic conditions, hypoxia inducible factors (HIFs) accumulate in the nucleus and bind to the hypoxia-response element to drive the transcription of their target genes. Therefore, hypoxia-induced thrombosis can be controlled directly by HIFs and their target genes (Gupta et al., 2017; Gupta et al., 2019). For instance, HIFs promote thrombosis by downregulating expression of protein S and TFPI and upregulating expression of procoagulant tissue factor and plasminogen activator inhibitor 1 (PAI-1) (Ahn et al., 2010; Cui et al., 2017). On the other hand, hypoxia can boost the release of Weibel Palade bodies and upregulate P-selectin expression in endothelial cells (Pinsky et al., 1996), as well as stimulate platelet activity through prethrombotic response and increase the production of prethrombotic factor or reduce that of antithrombotic factor in an HIF-independent manner (Tyagi et al., 2014; Gupta et al., 2019).

## Specific Pathogenic Mechanisms for Pulmonary Thromboembolism Associated With Deep Vein Thrombosis

In addition to the common pathogenic mechanisms underlying thrombosis, local anatomical characteristics, traumatic, pharmacological, and infectious triggers, as well as biochemical imbalance also contribute to the specific pathogenesis of PTE, *in situ* PAT, and CTEPH.

The specific mechanism responsible for PTE derived from DVT begins with Virchow’s triad: stagnation, plasma hypercoagulability, and endothelial injury at systemic level (Bagot and Arya, 2008). When the body is exposed to the systemic risk factors of thrombosis, the venous valve of the lower extremity become a major susceptible site to venous thrombosis due to its anatomical characteristic. Under normal circumstance, venous valves in the veins of lower extremities prevent blood reflux (Aird, 2007). As blood flows through venous valves, vortical stream behind the valve cusps attenuates blood stagnation in the valve pocket (Lurie et al., 2003). However, the blood in the valve pocket is more hypoxic and static than the main stream, hence generating a hypercoagulable microenvironment in the valve pocket (Aird, 2007). Especially in the patients with surgical anesthesia and long-term immobilization, reduced muscle activity slows venous blood flow and diminishes partial oxygen pressure (Hamer et al., 1981; Soo Hoo, 2013), promoting blood stasis and thrombosis in the venous valve pocket. Finally, the thrombus develops in the deep veins of lower extremeties. Triggerred by surgical operations, drug adminstration, or patient activities, the clot falls off, travels with circulation, reaches right atrium and ventricle, blooks the main body or branches of pulmonary arteries, and ends up with PTE (Soo Hoo, 2013; Xiao et al., 2018).

## Specific Pathogenic Mechanisms for *in situ* Pulmonary Artery Thrombosis

The main pathogenesis for *in situ* PAT is deemed as pulmonary local factors including pulmonary vascular endothelial cell dysfunction, hypoxia, and inflammation (Bennett et al., 2009; López and Chen, 2009).

Chest contusion and blast injury destroy pulmonary alveolar capillaries and blood-air barrier, leading to exudation into pulmonary interstitia (Ganie et al., 2013). Besides, the patients with pulmonary trauma tend to have atelectasis, which impairs gas exchange and deteriorates hypoxia. Then the substantially reduced alveolar oxygen partial pressure induces hypoxic vasoconstriction (Van Gent et al., 2014). On the other hand, at the early stage of thoracic trauma, inflammatory cells infiltrate into the damaged lung tissue and release inflammatory mediators, such as Interleukin-8 (IL-8) and Interleukin-6 (IL-6), resulting in elevated levels of these inflammatory mediators in alveoli (Hoth et al., 2006). Subsequently, the pulmonary trauma-induced hypoxia and inflammation activate endothelial cells (Van Hinsbergh, 2012), platelets (Lowenberg et al., 2010), and monocytes (Levi et al., 2006), all of which coordinate to cause *in situ* PAT.

Coronavirus disease 2019 (COVID-19) is a highly contagious and potentially fatal disease that has caused a pandemic. The autopsy of the patients who died from COVID-19 showed diffusive alveolar damage and infiltration of inflammatory cells, which are non-specific however, as the patients who died from acute respiratory distress syndrome and infection of other respiratory viruses exhibited the similar pathology (Kamel et al., 2020). Nonetheless, the researchers did find three distinctive pathogloical features in the lungs infected with severe acute respiratory syndrome coronavirus 2 (SARS-CoV-2). First, the cytokines were dramatically induced (Mehta et al., 2020) and pulmonary vascular endothelial cells severely injured by the virus infection. Secondly, the microthrombi of different ages were present throughout pulmonary microvasculature, implicating their local pulmonary origin. Thirdly, angiogenesis was promoted by upregulated expression of angiogenic factors (Ackermann et al., 2020). Therefore, COVID-19 pathology suggests that *in situ* PAT may be induced by pulmonary vascular endothelial injuries and the locally-produced proinflammatory cytokine storm (Gabrielli et al., 2020; Mandal et al., 2021). The pathogenic mechanism underlying the thrombogenicity of COVID-19 has been proposed. After SARS-CoV-2 binds to angiotensin converting enzyme 2, the cell surface receptor of the virus on type II pneumocytes and pulmonary vascular endothelial cells, the expression of angiotensin II upregulated in compensation (Fraga-Silva et al., 2010), which may elicit cytokine storm from activated inflammatory cells and endothelial cells through dysregulation of the rennin-angiotensin-aldosterone system (Mandal et al., 2021). The cytokine storm, particularly the upregulated expression of IL-6, subsequently leads to the injuries of alveoli and pulmonary vascular endothelial cells, turning the phenotype of the endothelial cells into pro-inflammatory and pro-thrombotic (Schieffer et al., 2000; Li et al., 2020). The inflammation and hypoxaemia may further amplify the vascular endothelial response and augment thrombus formation (Gupta et al., 2019). It is notable that COVID-19 does involve multiple organs, the abovementioned pathogenic mechanism underlying COVID-19 thrombogenicity can be applied at systemic level.

## Specific Pathogenic Mechanisms for chronic Thromboembolic Pulmonary Hypertension

CTEPH is a dual pulmonary vascular disease: obstruction of pulmonary arteries by unresolved thrombi and progressive remodeling of unobstructed vessels under increased pressure and shear stress (Simonneau et al., 2017; Yandrapalli et al., 2018) (Figure 2). The pathology of CTEPH corresponds to these two events. First, the organized thrombi comprised of collagens and fibrins entangled with debris of fibroblasts, lymphocytes, and macrophages attach to the arterial wall and form the lesion of “bands and webs”. Small and insufficient neovessels in the organized clots try to “recalnal” the blocked vessels with systemic circulation. Second, the plexiform lesions, the histological hallmark of PH, can be observed in the unobstructed pulmonary arterioles, indicating intimal hyperplasia and vascular remodeling (Lang et al., 2016). Several factors, including fibrinolytic abnormality, inflammation, angiogenesis, vascular remodeling, and *in situ* PAT, have been considered causing the CTEPH pathology (Yan et al., 2019).

The primary pathogenic factor of CTEPH is continuous insoluble thrombus in pulmonary vessels. Fibrinolysis is the initial stage of thrombus breakdown, followed by an inflammatory response that recruits neutrophils to continue decomposition. In the meanwhile, monocytes and endothelial progenitor cells are also recruited to promote clot reorganization and angiogenesis (Medrek and Safdar, 2016). Studies have demonstrated impairment in fibrinolytic system and deficiency in fibrin in the patients with CTEPH (Yan et al., 2019). For instance, plasma levels of tissue plasminogen activators and PAI-1 were significantly increased in CTEPH patients as compared with age-matched controls, yet no difference in enzymatic activity was detected between the two cohorts (Olman et al., 1992). Similarly, Lang et al. (1994) showed that the expression of PAI-1 protein and transcript was drastically upregulated in the endothelial cells and smooth muscle cells in the highly organized thrombus in comparison to the uninvolved areas in the pulmonary arteries of the CTEPH patients who had undergone pulmonary endarterectomy. In a recent study, Satoh et al. (2017) found that plasma level and pulmonary immunostaining intensity of thrombin-activatable fibrinolysis inhibitor were dramatically elevated in the patients with CTEPH as compared to the patients with lung cancer or pulmonary arterial hypertension. Furthermore, in a murine model of hypoxia-induced PH, ablation of the gene encoding TAFI ameliorated the phenotypes of PH, mitigated PAT, normalized plasma clot lysis time, and attenuated perivascular infiltration of macrophages and monocytes. Conversely, universal or liver-specific overexpression of TAFI exacerbated these parameters. These results implicate a crucial role of TAFI in the pathogenesis of CTEPH. Mechanistically, the plasma TAFI released from the liver binds to its binding partner thrombomodulin that is specifically upregulated in pulmonary artery endothelial cells. The TM-mediated enrichment and activation of TAFI specifically downregulate tight junction expression between the PAECs and lead to endothelial cell permeability, smooth muscle cell proliferation, and inflammatory cell infiltration. All of these events contribute to vascular remodeling and CTEPH (Satoh et al., 2017). Moreover, a genetic study has shown that the Thr312Ala mutation in the gene encoding fibrinogen protein may increase the risk of thrombosis and fibrinolytic resistance through altered homogenous cross linkage between fibrinogen α chains, thereby increasing the risk of CTEPH (Li et al., 2013).

In addition, transient inflammatory response is conducive to thrombolysis, however, the long-lasting one may exert the opposite effect. Studies have demonstrated that plasma levels of proinflammatory factors, including IL-6, IL-8, IL-10, interferon-γ, monocyte chemotactic protein-1, macrophage inflammatory protein-1α, and matrix metalloproteinase-9 were significantly elevated in CTEPH patients as compared with healthy controls (Zabini et al., 2014; Quarck et al., 2015).

Angiogenesis is crucial to recanalization and resolution of thrombus. The development of CTEPH may be associated with insufficient angiogenesis in thrombus (Alias et al., 2014). Quarck et al. (2015) proved that the plasma levels of vascular endothelial growth factor in CTEPH patients tended to be lower than healthy controls. Nonetheless, in the lung samples collected from CTEPH patients subjected to endarterectomy, the expression of angiostatic factors, such as platelet factor 4, collagen type I, and interferon-γ inducible 10 kD protein, was significantly upregulated as compared to those from healthy donors. Further, the angiostatic factors have been shown to disturb calcium homeostasis and induce endothelial cell dysfunction (Zabini et al., 2012). In addition, the high shear stress and pressure produced by redirected blood flow at unobstructed pulmonary vessels induce phenotypic changes and dysfunction of pulmonary vascular endothelial cells (Simonneau et al., 2017; Salibe-Filho et al., 2020). The endothelial cell dysfunction then stimulates secretion of inflammatory factors, such as fibroblast growth factor-2 and adhesion molecules, promoting proliferation of vascular smooth muscle cells and vascular remodeling (Mercier et al., 2017).

Finally, many CTEPH patients lack a history of acute PTE, and repeated embolization combined with ligation of pulmonary lobar artery failed to completely recapitulate CTEPH in animal models (Lang et al., 2013), thus for the cases of CTEPH without an acute PTE, it is possible that *in situ* PAT may initiate at the most susceptible site as a result of inflammation and imbalance between thrombosis and fibrinolysis, and then evolve to block the proximal and distal pulmonary arteries, leading to hypoperfusion and overflow of the blocked vessels and unblocked vessels, respectively. The unblocked vesels may be remodeled under excessive pressure and stress, eventually contributing to CTEPH (Egermayer and Peacock, 2000). However, this pathogenesis awaits further investigation and verification.

## Therapeutic Strategies

Research has revealed that in addition to the common pathogenic mechanisms, differential molecular pathogenesis does exist for different types of thrombosis. Therefore, specific preventive interventions and therapeutic strategies should be provided based on the distinct risk factors and pathogenesis of different thrombotic diseases. Furthermore, the interventions and therapeutics should not only manage the acute presentation, but also mitigate long-term sequela and reduce recurrence.

Three therapeutic strategies are suggested. First, for the thrombotic patient with a specific trigger, the trigger should be removed for the optimal efficacy of the current treatment and long-term survival with minimized recurrence. For example, the patients under the condition of infection, systemic inflammation, or high altitude hypoxia should first receive systemic anti-inflammatory or oxygen therapy for trigger removal (Aksu et al., 2012), and anticoagulation or surgical therapies ensue.

Second, for the thrombotic patients without an obvious trigger or with an irremovable trigger, anticoagulation is a must. For instance, although balloon pulmonary angioplasty (BPA) is an effective interventional modality for the patients with CTEPH, especially for the CTEPH patients with thrombi in the distal pulmonary arteries, the oral anticoagulant drugs such as warfarin, should be maintained lifetime to prevent recurrence of CTEPH following BPA (Panahi et al., 2021).

Third, mechanistic studies have identified key molecules and cell types in the coagulation cascade, which may subserve the molecular and cellular targets to counteract thrombosis in the long run. Anticoagulant drugs can be developed by downregulating P-selectin or inhibiting the interaction between P-selectin and PSGL-1 in the thrombotic diseases where vascular endothelial cells are activated but not injured (Furie and Furie, 2008). Moreover, microparticles play an important role in promoting thrombosis under inflammatory and malignant conditions, therefore the agent targeting microparticles need to be developed and used in combination with anti-inflammatory and anticancer drugs. Additionally, antiplatelet medicines, such as aspirin targeting thromboxane synthesis and clopidogrel targeting ADP receptor, can be used in the diseases incurring platelet activation (Wu and Matijevic-Aleksic, 2005).

## Conclusion

For a long time, the thrombi of PTE have been considered originated from deep veins of lower extremities, however, accumulating evidence reveals that pulmonary thrombi may be generated *in situ* following chest trauma, pulmonary diseases, and systemic inflammatory and immunological disorders. The currently known mechanism underlying *in situ* PAT originates from the local hypoxic and inflammatory milieu, which then induces pulmonary vascular endothelial cell dysfunctions following injury, diseases, and drug interventions and subsequently leads to imbalance between thrombosis and fibrinolysis. The thrombi of different sources, either deep veins in lower limbs or pulmonary vasculature *in situ*, if not resolved in lung in a timely manner, may cause CTEPH.

Collectively, we suggest that a new group of diseases involving PH, “pulmonary artery thrombotic diseases”, be proposed. This group refers to all the thrombotic events that may occour in pulmonary artery, and currently can include three distinct diseases, PTE, *in situ* PAT, and CTEPH. The inter-relationship among the three diseases is complicated. PTE and *in situ* PAT can occur independently or coexist; either PTE or *in situ* PAT or both, if not appropriately managed in a timely manner, may progress to CTEPH.
